# Distal Radius Fracture with Dorsal Angulation

**DOI:** 10.52965/001c.125163

**Published:** 2024-10-31

**Authors:** Ronith Bokkisam, Nofel Iftikhar, Latha Ganti

**Affiliations:** 1 Washington University; 2 University of Florida; 3 Orlando College of Osteopathic Medicine

**Keywords:** distal radius fracture, radial fracture angulation, FOOSH

## Abstract

Distal radial fractures (DRFs) are prevalent injuries and represent one of the most common fractures seen in the Emergency Department (ED). DRFs often result from falls on an outstretched hand (FOOSH). This case study details a 64-year-old female who sustained a distal radial fracture with dorsal angulations (DRFDA) from ice skating, confirmed via radiographic evaluation. Initial treatment involved immobilization, pain management, and follow-up with orthopedic specialists. Proper diagnosis and management are essential to prevent long-term functional impairments, with treatment ranging from casting to surgical intervention based on fracture severity.

## Introduction

Distal radius fractures constitute common injuries treated in the ED, representing over 640,000 cases treated in the ED annually. Defined by a fracture in the radius bone near the wrist, DRFs make up approximately 17.5% of all fractures in adults and 20% of all fractures treated in the ED.^1^ DRFs, in regards to subcategorization, may include Dorsal, Torus/Buckle, Die-punch, Greenstick, Smith’s, and isolated radial shaft fractures.^2^ DRFs occur near the end of the radius, where the fractured bone becomes displaced and the distal region of the bone shifts axes and points off in a different direction.^3,4^ DRFs are most commonly a result of falling on an outstretched hand, as observed in the presenting case, but can more generally be attributed to any form of high energy trauma (HET) to the wrist. DRF cases are represented, most commonly, via decreased wrist mobility and mild swelling.^5^ In this report, the authors describe a common case of DRF and report the treatment provided.

## Case Presentation

**Figure 1.**
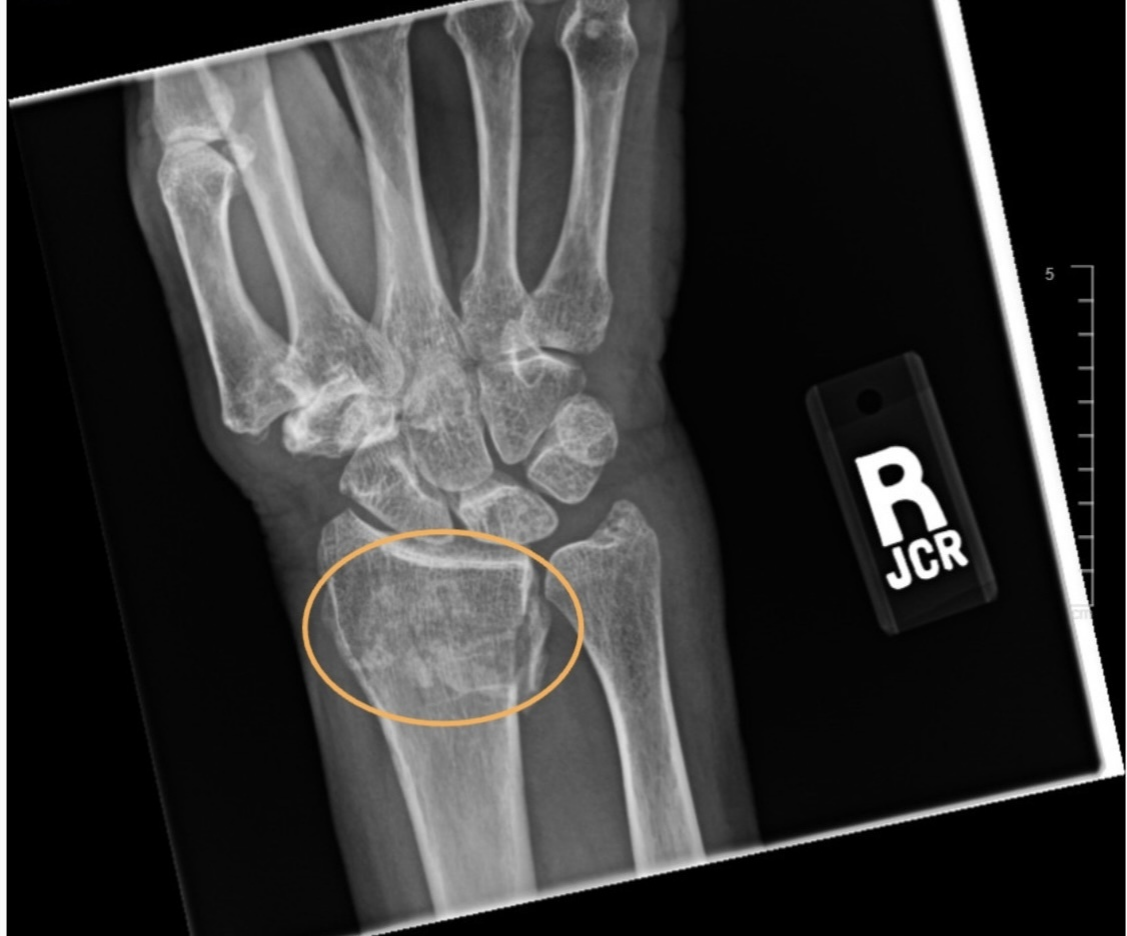
AP view wrist radiograph with the DRF circled in orange.

**Figure 2.**
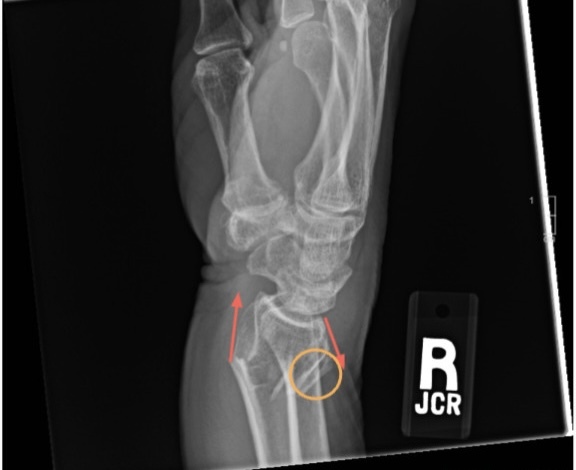
Lateral view wrist radiograph demonstrating a DRF with dorsal angulation, highlighted by an orange circle. The red arrows highlight the altered, non-parallel directions of the fractured bones.

A 64-year-old female patient presents to the ED, complaining of wrist pain, with visible guarding of the area. Earlier in the day, while ice skating, the patient, in an effort to break her fall, outstretched her hand and fell onto her wrist, resulting in the fracture. The patient’s vitals were measured as normal in regards to $O_{2}$ saturation (on room air), blood pressure, mean arterial pressure, temperature, pulse, and respiratory rate. Following an initial physical examination, soft tissue swelling was observed in the right/left wrist, the area of impact regarding the injury. No further injuries were observed and the patient was observed as alert and responded to stimuli. Although, as determined from the initial physical examination and the signs and symptoms observed, a wrist fracture was likely, the patient was sent to radiology to confirm the suspected finding, classify the injury further, and observe any other injuries overlooked by the physical examination. The x-ray featured an obvious DRF indicated by a clear fracture, displacement of radial bone, and angular distortion (Figures 1 & 2).

Immediate treatment for the patient in the ED was limited, but included stabilizing the injury via a sling splint.^6^ The patient was also scheduled to follow up with orthopedics for the following day to cast the injury, if needed. The patient was told to avoid any unneeded physical activity, ice the injury to avoid swelling, take painkillers if pain became severe, and visit the ED if needed.^7^

## Discussion

**Figure 3.**
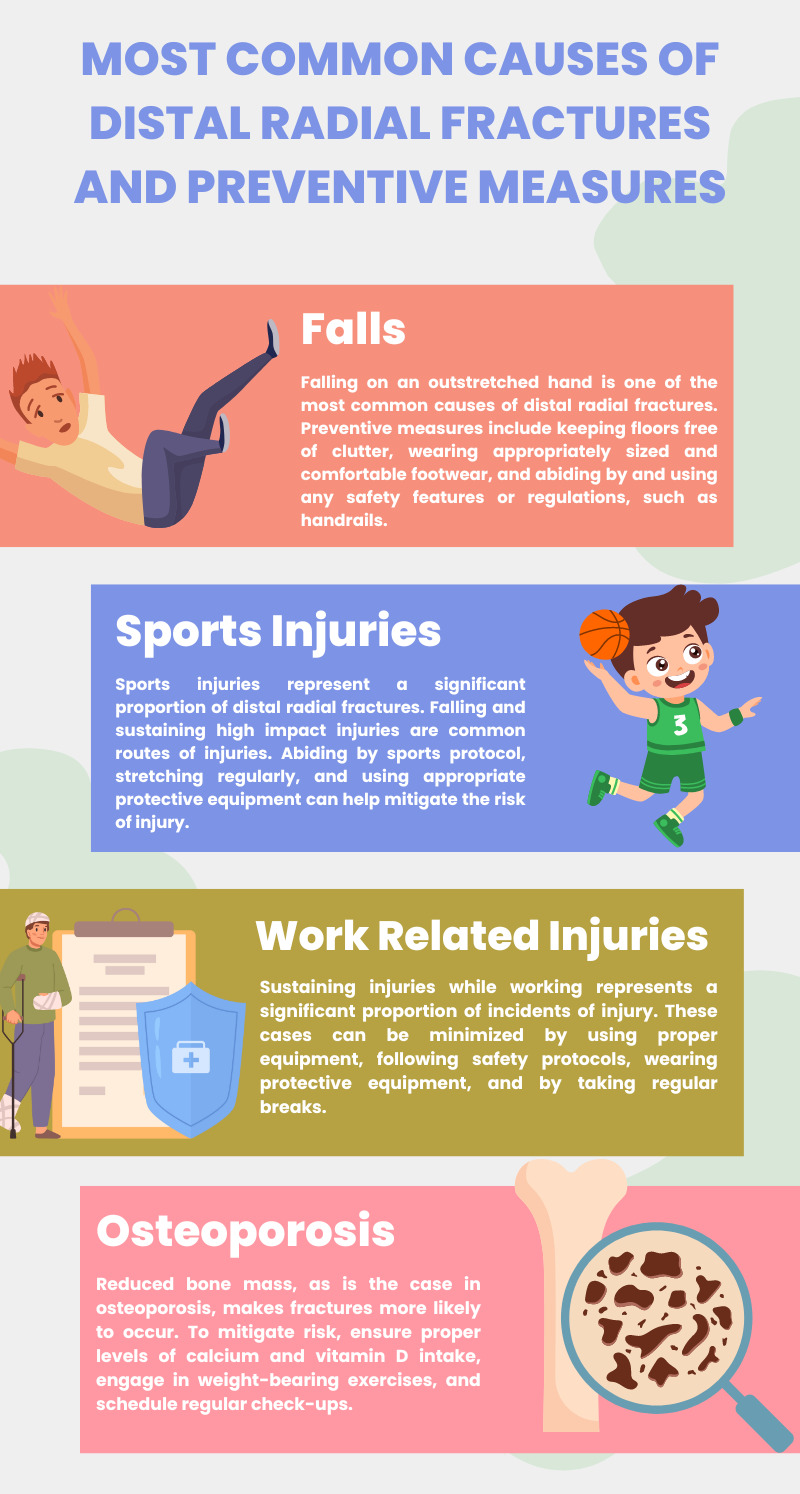
Infographic summarizing common causes and preventative measures for DRF injuries.

The case presented demonstrated a common “fall on an outstretched hand” (FOOSH) injury, which caused the fracture in the presenting case. FOOSH injuries lead to a maximum amount of force being applied to the wrist, causing DRFs.^8^ These types of injuries are most prevalent in younger individuals, who may participate in sports and other HET-inclined activities, and the elderly, who are more susceptible to falls, due to age-related physical and neurological decline, and fractures, as a result of osteoporotic changes and bone mass reduction.^9^ Other common causes of DRFs include, work-related injuries and motor vehicular accidents (Figure 3). Common symptoms of DRF cases include wrist pain, wrist swelling, loss of function, decreased mobility and strength, and decreased range of motion.^10^ Unaddressed, this angulation can result in a dramatic loss of function. Proper alignment of the wrist and forearm is critical to the maintenance of range of motion and strength.^11^

A type of FOOSH injury, DRFs are diagnosed based on physical examination and plain radiography, which is used to further clarify the location and extent of the fracture, thus dictating the treatment provided. In x-rays, DRFs commonly present with dorsal angulation of the fractured distal fragment. Radiographic assessment aids, in conjugation with physician physical examination, aid in preoperative planning and long-term healing efforts.^12^ Depending on the severity of the injury, treatment plans may differ. Initial treatment of DRFs include splinting the wrist, icing the injured area to prevent swelling, and the administration of painkillers, if needed. Additionally, follow up consultations are usually scheduled with orthopedic specialists. Based on radiographic findings and orthopedic consultations, casts may be applied, and in extreme cases surgical intervention may be necessary to correct the fracture.^13^ Definitive treatment depends on a number of factors, with the most common being: severity of fracture, displacement of fractured bone, and lifestyle and age of patient. In most cases, non-operative treatment with casting is the preferred choice for non-displaced fractures. However, for more severe fractures may necessitate open reduction and internal fixation to help realign and stabilize.^14^ Additionally, if necessary, surgical intervention by means of percutaneous pinning, and internal fixation using plates or screws, and closed reductions may be utilized durinrg the process.

## Conclusion

The case presented illustrated a common injury mechanism for DRF, emphasizing the importance of accurate diagnosis and appropriate initial management in the ED. Immobilization with a splint, pain management, and prompt follow-up with orthopedic specialists are crucial steps in ensuring effective treatment and recovery. Proper alignment and stabilization of the fracture are essential to maintain wrist function and prevent long-term complications. Given the prevalence of such injuries, particularly among the elderly and active individuals, continued research and education on optimal management strategies are necessary to improve patient outcomes and reduce the incidence of debilitating complications.
